# RETRACTED: Tsatali et al. Normative Data for the D-KEFS Tower Test in Greek Adult Population Between 20 and 85 Years Old. *Brain Sci.* 2025, *15*, 278

**DOI:** 10.3390/brainsci16050517

**Published:** 2026-05-13

**Authors:** Marianna Tsatali, Despina Eleftheriadou, Nikoleta Palla, Magda Tsolaki, Despina Moraitou

**Affiliations:** 1 Laboratory of Psychology, Department of Cognition, Brain and Behavior, School of Psychology, Aristotle University of Thessaloniki (AUTh), 54124 Thessaloniki, Greecedemorait@psy.auth.gr (D.M.); 2 Department of Psychology, School of Humanities and Social Sciences, University of Western Macedonia, 50100 Kozani, Greece; 3 Greek Association of Alzheimer’s Disease and Related Disorders (GAADRD), 54248 Thessaloniki, Greece; 4 Lab of Neurodegenerative Diseases, Center for Interdisciplinary Research and Innovation, Aristotle University of Thessaloniki (CIRI-AUTh), 54124 Thessaloniki, Greece

The journal retracts and removes the article, “Normative Data for the D-KEFS Tower Test in Greek Adult Population Between 20 and 85 Years Old” [1], cited above.

Following publication, the authors informed the Editorial Office about a concern relating permission to publish material presented in this study [1].

In accordance with the journal’s standard procedures, the Editorial Office and Editorial Board conducted an investigation that confirmed that appropriate permission to publish material related to the D-KEFS test materials and Greek D-KEFS norms had not been gained prior to publication. The authors cooperated fully with the Editorial Office throughout the investigation and submitted a revised version of the manuscript. However, the Editorial Board determined that the extent of the necessary revisions exceeded what could be addressed through a standard correction. For this reason, Editorial Board have decided to retract the article [1], which will also be removed in order to comply with intellectual property rights, and invite the authors to submit a revised version using an authorized test, which will be considered as a new submission and subject to the journal’s standard peer-review process.

As such, the title, author information and this retraction note will remain permanently available, and the article itself has been removed. This information has also been transmitted to relevant indexing organizations.

This retraction was approved by the Editor-in-Chief of the *Brain Sciences* journal.

Authors agreed to the retraction.

## References

[1] Tsatali M., Eleftheriadou D., Palla N., Tsolaki M., Moraitou D. (2025). RETRACTED: Normative Data for the D-KEFS Tower Test in Greek Adult Population Between 20 and 85 Years Old. Brain Sci..

